# A virulent milRNA of *Fusarium oxysporum* f. sp. *cubense* impairs plant resistance by targeting banana AP2 transcription factor coding gene *MaPTI6L*

**DOI:** 10.1093/hr/uhae361

**Published:** 2024-12-28

**Authors:** Jiaqi Zhong, Junjian Situ, Chengcheng He, Jiahui He, Guanghui Kong, Huaping Li, Zide Jiang, Minhui Li

**Affiliations:** College of Plant Protection, South China Agricultural University, Guangzhou, GD 510642, China; College of Plant Protection, South China Agricultural University, Guangzhou, GD 510642, China; College of Plant Protection, South China Agricultural University, Guangzhou, GD 510642, China; College of Plant Protection, South China Agricultural University, Guangzhou, GD 510642, China; College of Plant Protection, South China Agricultural University, Guangzhou, GD 510642, China; Guangdong Province Key Laboratory of Microbial Signals and Disease Control, South China Agricultural University, Wushan Road, Tianhe District, Guangzhou, GD 510642, China; College of Plant Protection, South China Agricultural University, Guangzhou, GD 510642, China; Guangdong Province Key Laboratory of Microbial Signals and Disease Control, South China Agricultural University, Wushan Road, Tianhe District, Guangzhou, GD 510642, China; College of Plant Protection, South China Agricultural University, Guangzhou, GD 510642, China; Guangdong Province Key Laboratory of Microbial Signals and Disease Control, South China Agricultural University, Wushan Road, Tianhe District, Guangzhou, GD 510642, China; College of Plant Protection, South China Agricultural University, Guangzhou, GD 510642, China; Guangdong Province Key Laboratory of Microbial Signals and Disease Control, South China Agricultural University, Wushan Road, Tianhe District, Guangzhou, GD 510642, China

## Abstract

Fungi produce microRNA-like RNAs (milRNAs) with functional importance in various biological processes. Our previous research identified a new milRNA *Foc*-milR87 from *Fusarium oxysporum* f. sp. *cubense*, which contributes to fungal virulence by targeting the pathogen glycosyl hydrolase encoding gene. However, the potential roles of fungal milRNAs in interactions with hosts are not well understood. This study demonstrated that *Foc*-milR87 specifically suppressed the expression of *MaPTI6L*, a pathogenesis-related gene that encodes a transcriptional activator in the banana (*Musa acuminata* Cavendish group cv. ‘Baxi Jiao’) genome, by targeting the 3'untranslated region (UTR) of *MaPTI6L*. Transient overexpression of MaPTI6L activated plant defense responses that depend on its nuclear localization, yet co-expression with *Foc*-milR87 attenuated these responses. MaPTI6L enhanced plant resistance by promoting transcription of the salicylic acid signaling pathway marker gene *MaEDS1*. Sequence analysis of the *MaPTI6L* gene in 19 banana varieties, particularly those resistant to Fusarium wilt, uncovered single nucleotide polymorphisms (SNPs) at *Foc*-milR87 target sites. Experimental validation showed that these SNPs significantly reduce the microRNA's ability to suppress target gene expression. Our findings reveal that *Foc*-milR87 plays an important role in impairing plant resistance by targeting MaPTI6L mRNA and reducing *MaEDS1* transcription during the early infection stage, suggesting the 3'UTR of *MaPTI6L* as a promising target for genome editing in generation of disease-resistant banana cultivars.

## Introduction

Bananas (*Musa* spp.) are widely cultivated in tropical or subtropical countries and regions and are considered as one of the world’s most valuable primary agricultural commodities [1]. However, banana Fusarium wilt (BFW) caused by the fungus pathogen *Fusarium oxysporum* f. sp. *cubense* (*Foc*) is severely threatening the global banana production and restricting the international banana trade [2, 3] So far, tropical race 4 (TR4) widely distributed throughout the world and is regarded as the most destructive banana pathogen among the four reported races of *Foc* [4]. Currently, the main commercial cultivar, Cavendish, which previously spared banana from extinction by *Foc* race1 in the 1960s is now susceptible to TR4. BFW management has become increasingly challenging after the discovery of TR4 [5]. Disease-resistant cultivars are urgently required for banana producers to replace existing Cavendish bananas; however, only relative resistant somaclones have been created by prolonged tissue culture [6, 7]. Recently, a few resistance genes have been introduced into Cavendish plants, and transgenic plants impart TR4 resistance in the field [8, 9]. However, it is impossible to predict whether or how long it will take for these transgenic bananas to be approved [10]. Therefore, elucidating the interaction mechanism between *Foc* and bananas would provide more genetic resources for the BFW control.

Fungal microRNA-like RNAs (milRNAs) are a type of endogenous noncoding small RNA (sRNA) identified initially in the model fungus *Neurospora crassa* and are biosynthesized from single-stranded RNA precursors characterized by distinctive stem-loop secondary structures [11]. An increasing number of milRNAs have been discovered to target genes in both host plants and pathogens, and they may play important roles in diverse biological processes [12, 13]. These milRNAs often function as effectors in plant pathogenic fungi to degrade the mRNA of host defense-related gene. For example, a *Botrytis cinerea* sRNA effector, *Bc-*siR37, was reported to target *AtWRKY7*, *AtPMR6*, and *AtFEI2* genes to suppress defense mechanisms in *Arabidopsis* [14]. *Vm*-milR1 from *Valsa mali* was discovered to act as an sRNA effector to suppress host receptor-like kinase genes and thereby enhance the infection of pathogen [15]. In-depth research on fungal milRNAs would therefore assist in the identification of host immunity genes that could be used to develop disease-resistant plants. A recent study on *F. oxysporum* f. sp. lycopersici (*Fol*) highlighted the critical roles of milRNA during *F. oxysporum*–host interaction [16]. However, the potential roles of the milRNAs from *Foc* in interactions between the pathogen and banana are not well understood.

The APETALA2/ethylene-responsive element binding factors (AP2/ERF) transcription factors are mainly found in plants, regulating many biological processes, such as plant morphogenesis, responses to abiotic and biotic stresses, hormone signal transduction, and metabolite regulation [17–20]. The rice AP2/ERF transcription factor, OsDREB2B, physically interacts with OsSRO1c and responds to low temperature through dynamic liquid–liquid phase transitions and regulates key cold-response genes [21]. OsLG3 fine-tunes the progression of rice leaf senescence by suppressing abscisic acid (ABA) signaling and simultaneously activating ROS scavenging [22]. ERF68 protein of tomato was found to promote the expression of defense genes and plant cell death, culminating in the incompatible interactions between tomato and *Xanthomonas* spp. [23]. Moreover, the GmERF113 was also demonstrated to enhance soybeans resistance against *Phytophthora sojae* [24]. Although these findings underscore the diverse role of AP2/ERF transcription factors in biological processes, their functions in banana disease resistance remains largely unknown.

Our previous study found that milRNA, *Foc-*milR87, acts as a virulence factor by silencing the pathogen glycosyl hydrolase-coding gene and enables the pathogen to evade the activation of host defense responses, eventually facilitating *Foc* infection [25]. In this study, to further investigate how *Foc-*milR87 undermines the plant defense responses and bolsters the virulence of pathogen, we predicted and confirmed the target gene of *Foc-*milR87 in the genome of wild banana (*M. acuminata* subsp*. malaccensis*). Through heterologous expression of *Foc-*milR87 and its target gene *MaPTI6L* in *Nicotiana benthamiana* leaves, we proved that *Foc-*milR87 can curtail the plant defense and facilitate pathogenic infection by silencing *MaPTI6L*. We report that *Foc-*milR87 derived from *F. oxysporum* f. sp. *cubense* targets *MaPTI6L* to suppress host plant immunity, which helps elucidate the interactions between the fungal pathogen *Foc* and banana hosts.

## Results

### *Foc-*milR87 is highly conserved in *F. oxysporum*

**Figure 1:**
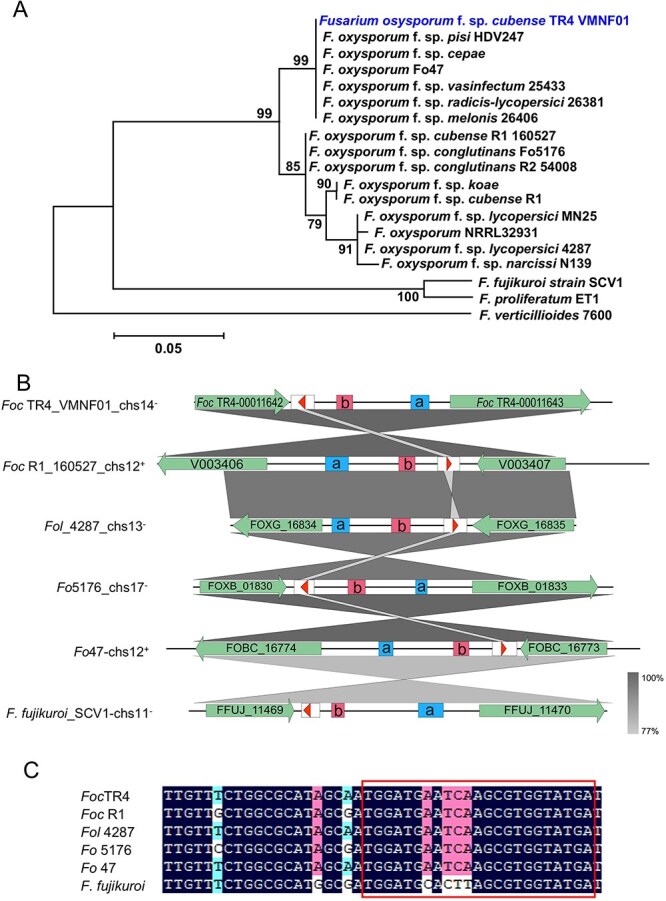
*Foc*-milR87 is highly conserved in *F. oxysporum*. (A) The maximum likelihood tree was constructed with using MEGA X. Bootstrap percentages out of 1000 replications are shown at branch points. *Foc*-milR87 is shown as the first taxon. (B) Synteny of loci of *Foc*-milR87 in *F. oxysporum* and *F. fujikuroi*. The long arrows represent protein coding regions. The boxes with the small arrows represent precursors of milRNAs, while the small arrows represent mature milRNAs; a and b represent tRNAs and rRNAs, respectively (the deeper the gray, the greater the similarity between the two adjacent sequence groups). (C) The outlined sequence alignment represents the mature *Foc*-milR87 in *Fusarium* spp.

Based on our previous research, *Foc*-milR87 is a key pathogenicity-related milRNA that contributes to virulence during the early stages of infection (0–96 h post-inoculation, hpi) by targeting the *Foc* own glycosyl hydrolase gene [25]. Using the precursor gene sequence of *Foc*-milR87 as a query, we performed the default BLASTN searches against the genomic sequences in the ENSEMBL fungal database (https://fungi.ensembl.org/Tools/Blast?tl=Kl7PcOATzPbMf4XF-21631558), and retained homologs longer than 50 bp for phylogenetic analysis. These searches identified a total of 19 fungal milRNA genes, including 16 from various *F. oxysporum* forma speciales and three from *F. fujikuro*, *F. proliferatum,* and *F. verticillium*. The phylogenetic analysis revealed that the *Foc*-milR87 precursor gene and its homologs from other *F. oxysporum* forma speciales were closely clustered with a high similarity of 94%–100%, and they were distantly clustered with homologs from *F. fujikuroi* and *F. proliferatum* in one group, while homologs from *F. verticillium* was served as the outgroup (Fig. 1A). All homologs of *Foc*-milR87 were located in the intergenic regions and were adjacent to ribosomal RNAs as shown in Fig. 1B. Except for orientation, all loci of homologs and their adjacent genes were the same (Fig. 1B). The sequences of all homologous milRNAs from *F. oxysporum* shown in Fig. 1C are same as that of *Foc*-milR87. The above results indicated that *Foc*-milR87 is highly conserved and specific in publicly available genomes of the *F. oxysporum* species complex.

### *Foc*-milR87 specifically targets the 3'UTR of *MaPTI6L* during infection by *Foc*

**Figure 2:**
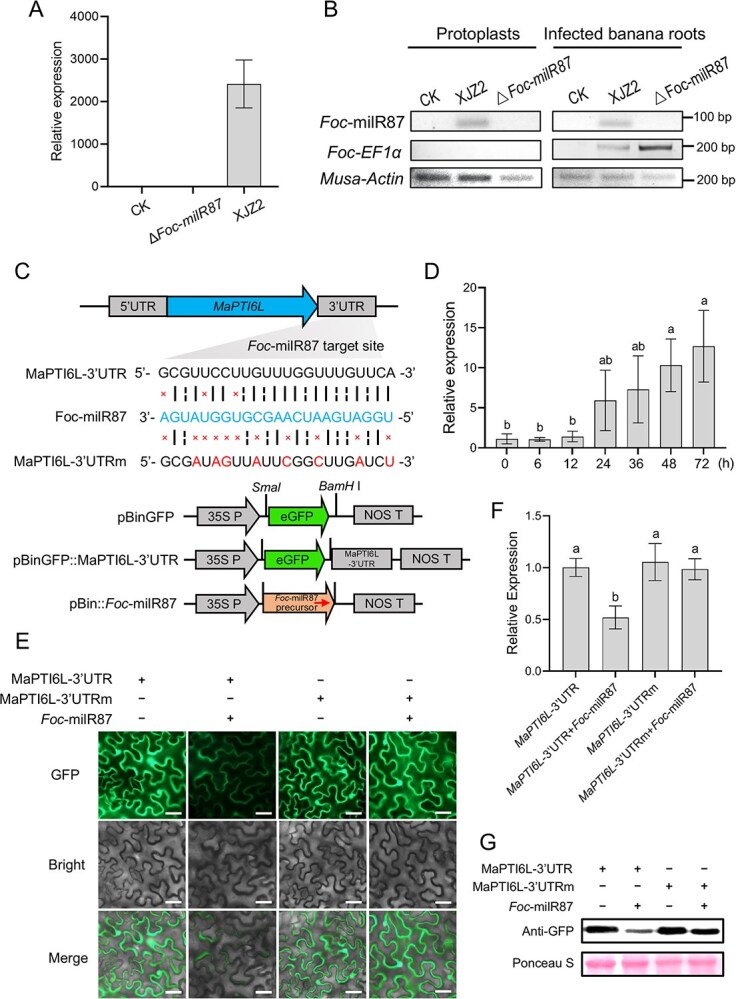
*Foc*-milR87 translocated from the pathogen to the host plant and suppressed the expression of MaPTI6L in *Nicotiana benthamiana*. (A) qRT-PCR analysis of *Foc*-milR87 in banana roots protoplasts. Total RNAs were extracted from banana roots and banana root protoplasts inoculated with *Foc* 36 h prior. CK is the H_2_O treatment. Relative expression levels of *Foc*-milR87 were calculated by 2^-ΔΔCt^ method using the *Actin* gene of banana as internal control. Error bars indicate Standard deviations (SD); *n* = 3. (B) PCR analysis of *Foc*-milR87 in banana root protoplasts. PCR amplicons viewed on 2% agarose gels. (C) Schematic diagram of constructs with the wild-type or mutated 3'UTR of *MaPTI6L* and *Foc*-milR87 construct. The small arrow represents mature milRNA of *Foc*-milR87. (D) Expression of *Foc*-milR87 in *N. benthamiana* was detected after agro-infiltration with construct pBin::*Foc*-milR87 at different time points by qRT-PCR. And snRNA (U6) of *N. benthamiana* was used as internal control. The expression value of 0 h was set to 1. Different letters indicate significant differences at α = 0.01. (E) Confocal images after co-infiltration of pBinGFP::MaPTI6-3'UTR or mutated pBinGFP::MaPTI6-3'UTRm and pBin::*Foc*-milR87 in *N. benthamiana*. Confocal images were taken at 48 h after agro-infiltration. Bar, 50 μm. (F) Transcript levels of GFP gene were analyzed by qRT-PCR. The expression value of GFP after pBinGFP::MaPTI6-3'UTR treatment was set to 1 and *NbEF1α* was used as internal control. Duncan’s multiple range test was applied to distinguish means; bars with identical letters on top show no significant differences at α = 0.01. Error bars stand for S.D.; *n* = 4. (G) Western blot analysis of GFP intensity was performed. Same amounts of proteins were loaded. Anti-GFP antibodies were adopted for western blot analysis. Protein loading is shown by Ponceau S staining. The co-expression experiment was independently repeated thrice with analogous results

As *Foc*-milR87 was significantly induced during the initial infection stage (Fig. S1) [25], we hypothesized that this milRNA was capable of interfering with plant immunity. To verify that *Foc*-milR87 was exported from *Foc* into banana cells, we inoculated banana roots with *Foc* conidia followed by protoplast preparation and quantitative real time-PCR (qRT-PCR) analysis. As shown in Fig. 2A, *Foc-*milR87 was detected in protoplasts derived from banana roots infected with the WT strain XJZ2, but not by the *Foc*-milR87 knockout mutant Δ*Foc-milR87.* To further verify the presence of *Foc-*milR87 in plant cells, we amplified the *Foc*-milR87 in the infected banana roots and protoplasts, separately by RT-PCR. The results showed that *Foc*-milR87 could be detected in the banana roots and protoplasts derived from banana roots infected with the WT strain XJZ2 (Fig. 2B)*.* However, no PCR amplicon of *Foc*-milR87 was detected in either banana roots or protoplasts derived from banana roots infected with Δ*Foc-milR87* (Fig. 2B). Simultaneously, *EF1α* of *Foc*, as a negative control, was not detected in protoplasts nor in non-inoculated banana roots, while *Actin* of *Musa* was detected in both samples. These results suggested that *Foc*-milR87 was secreted by *F. oxysporum* and indeed translocated into plant cells.

Next, we predicted the target genes from the genome of the wild banana (*M. acuminata* AAA group cv. ‘Baxi Jiao’) using the online software psRNATarget, and 47 potential candidates were selected for target gene identification. Expression of the candidates in response to infection by the WT and the Δ*Foc*-milR87 mutant of *Foc* TR4 was evaluated by qRT-PCR. At the early stages of infection, a predicted pathogenesis-related gene, *MaPTI6L*, encoding transcriptional activator PTI6-like protein from the host was induced by infection with the Δ*Foc*-milR87 mutant to a higher level than that induced by the WT (Fig. S2). Therefore, *MaPTI6L* was finally selected for target site identification of *Foc*-milR87.

The predicted targeted site of *Foc*-milR87 was located in the 3'UTR of *MaPTI6L* (Fig. 2C). We then generated a construct by adding the 3'UTR region right after the GFP encoding sequence (Fig. 2C). A point-mutated UTR that could not be paired with *Foc*-milR87, named MaPTI6L-UTRm, was also integrated with GFP (Fig. 2C). Through transient expression of *Foc*-milR87 in *N. benthamiana* leaves, the milRNA was detected and their expression was gradually increased after infection by *Agrobacterium tumefaciens* carrying the *Foc*-milR87 construct (Fig. 2D). To verify whether the *MaPTI6L* 3'UTR could be targeted by *Foc*-milR87, we built constructs with WT or mutated 3'UTR integrated with GFP, which were transiently co-expressed with the milRNA in *N. benthamiana* leaves. The GFP signal was evident when GFP-UTR or GFP-UTRm was expressed alone, but significantly suppressed by co-expressed with GFP-UTR and *Foc*-milR87 at two days post-injection of *A. tumefaciens*. As expected, the GFP signal was not attenuated when co-expression with GFP-UTRm and *Foc*-milR87 (Fig. 2E). Correspondingly, transcript levels and protein abundance of GFP, expressed alone or co-expressed with *Foc*-milR87, were consistent with that of GFP signal detection (Fig. 2F and G). Similarly, the mRNA abundance of *MaPTI6L* also decreased when MaPTI6L (CDS + 3'UTR) was co-expressed with *Foc*-milR87 (Fig. S3). Collectively, these data demonstrated that *MaPTI6L* is targeted by *Foc*-milR87 at the 3'UTR in a sequence-specific manner.

### Expression of MaPTI6L activates immune responses in *N. benthamiana*

In the genome of wild banana, 12 genes were annotated as *PTI6* or *PTI6L*. Among them, *MaPTI6L* encodes a protein (Accession No.: XP_009390940.1) of 300 amino acids, which contains an AP2 DNA binding domain and a nuclear localization signal at 117–120 aa (Fig. S4A). The BLASTP search against the NCBI database indicated that MaPTI6L had the highest similarity (91.64%) to a hypothetical protein of *Ensete ventricosum*, which also is a large evergreen arborescent herb and belongs to the family Musaceae. Phylogenetic analysis showed that proteins annotated as PTI6 in the banana genome were divided into two independent clusters. Eight proteins named as MaPTI6 were clustered with tomato SlPTI6 (a Pto-interacting protein) [26] in one branch. The other cluster was composed of the remaining four MaPTI6L proteins including the target of *Foc*-milR87 and some homologous proteins from plants, suggesting that the MaPTI6L protein targeted by *Foc*-milR87 was phylogenetically independent from SlPTI6 identified in tomato (Fig. S4B).

**Figure 3:**
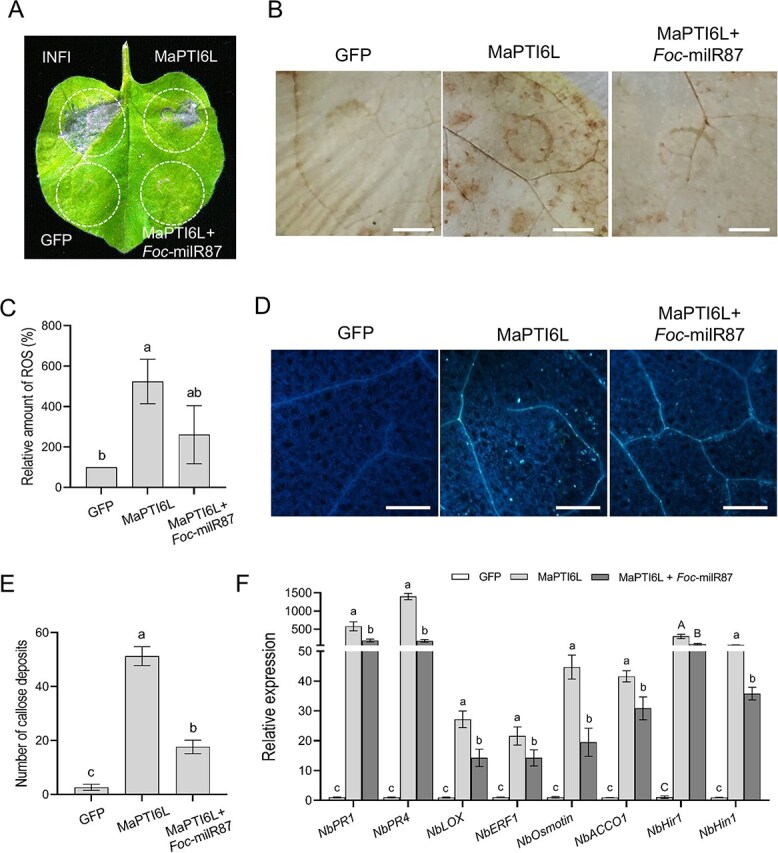
Expression of MaPTI6L activates plant immune responses and enhances multiple defense signaling pathways in *N. benthamiana*. (A) Cell death triggered by MaPTI6L in a *N. benthamiana* leaf. The leaf was infiltrated with *A. tumefaciens* carrying pBinGFP::MaPTI6L, and photographed at 5 days post agro-infiltration (dpa). GFP and INF1 were used as negative and positive control, respectively. (B and C) *N. benthamiana* overexpressed with MaPTI6L showed increased ROS accumulation at 2 dpa. GFP served as the negative control. Bar, 50 μm. ROS quantification was performed using ImageJ software. Error bars represent SD; *n* = 10. Duncan’s multiple range test was employed to separate means and bars topped by the same letter indicating no significant differences at a significance level of α = 0.01. (D and E) *N. benthamiana* overexpressed with MaPTI6L showed increased callose deposition at 2 dpa. Bar, 200 μm. Quantification of callose deposition in each picture using ImageJ software. Error bars indicate SD; *n* = 10. Duncan’s multiple range test was used to separate means and bars with the same letter on top are not significantly different at α = 0.01. (F) MaPTI6L overexpression enhances the expression levels of defense-related genes in *N. benthamiana*. The constitutively expressed gene *NbEF1α* was used as internal reference. Error bars indicate SD; *n* = 3. Duncan’s multiple range test was used to separate means. Bars with the same capital letter on top are not significantly different at α = 0.05, while bars with the same lowercase letter on top are not significantly different at α = 0.01. The experiment was repeated at least three times

To test whether MaPTI6L could activate plant immune responses, we transiently expressed MaPTI6L (CDS + 3'UTR, the same below) in *N. benthamiana* followed by the analysis of the local hypersensitive response (HR), reactive oxygen species (ROS) accumulation, and callose deposition. The results showed that transient expression of MaPTI6L caused chlorosis accompanied by slight cell death in *N. benthamiana* leaves at 48 h post-agroinfiltration (hpa; Fig. 3A). When MaPTI6L and *Foc*-milR87 were co-expressed, no leaf chlorosis or cell death occurred (Fig. 3A). Plants can accumulate anti-pathogen molecules such as ROS to defend against pathogens or to activate downstream immune responses during pathogen infection [27]. Overexpression of MaPTI6L significantly boosted ROS accumulation compared to the negative control, GFP, in *N. benthamiana* leaves at 48 hpa, but co-expression of MaPTI6L and *Foc-*milR87 reduced ROS accumulation (Fig. 3B and C). Callose is a β-1,3- glucan polysaccharide that deposits at the edge of plant sieve pores, generally existing outside the cell wall, enhancing cell wall physical strength, and helping prevent invasion of pathogens [28, 29]. Similarly, compared with the negative control, GFP, overexpression of MaPTI6L significantly increased callose deposition; however, when *Foc*-milR87 and MaPTI6L were co-expressed, the quantity of callose deposition was decreased but still more than that of the control (Fig. 3D and E). These results indicate that MaPTI6L positively activated plant immune responses, which were suppressed by co-expression with *Foc*-milR87, likely due to inhibition of MaPTI6L expression by *Foc-*milR87.

To further investigate whether the expression of MaPTI6L is related to activation of the plant defense signaling pathways in *N. benthamiana*, the expression of defense-related genes was monitored by qRT-PCR. The results confirmed that transient expression of MaPTI6L induced the expression of a large number of resistant marker genes involved in phytohormone signaling pathways, such as: salicylic acid (SA) pathway marker genes *NbPR1* and *NbPR4*; jasmonic acid (JA) pathway marker gene *NbLOX*; ethylene-related genes *NbERF1*, *NbOsmotin* and *NbACCO1*; as well as the HR related genes, *NbHir1* and *NbHin1* (Fig. 3F).

### MaPTI6L contributes to plants resistance against fungal pathogens

**Figure 4:**
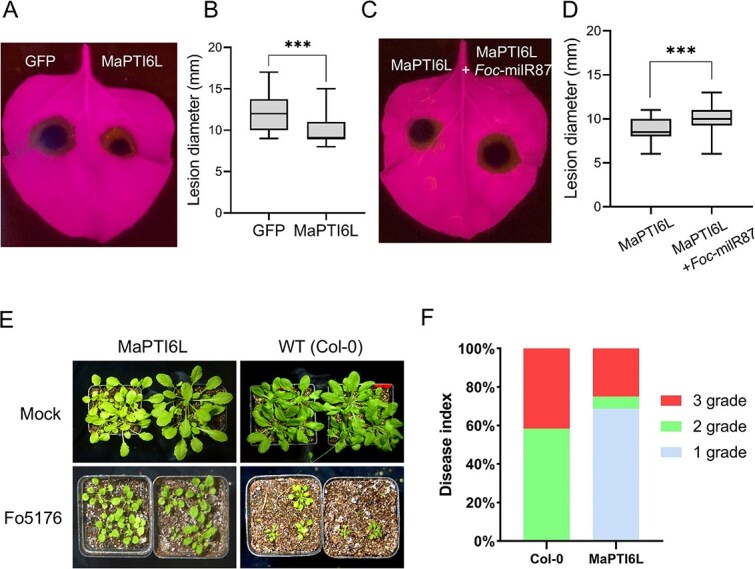
MaPTI6L confers resistance to fungal infection. (A and B) Overexpression of MaPTI6L confers resistance to *A. alternata* in *N. benthamiana* leaves. *A. alternata* was inoculated onto the leaf and photographed at 5 dpa. (C and D) Silencing *MaPTI6L* by *Foc*-milR87 curtails plant resistance to *A. alternata*. Photographs were taken at 5 days post *A. alternata* inoculation. In B and D, the error bars represent maximum and minimum values. Center line,median; box limits, 25th and 75th percentiles; *n*≥12. *** *P* < 0.01 (Student’s *t*-test). (E and F) The *Arabidopsis* overexpressing MaPTI6L exhibited enhanced resistance to Fo5176. Leaves from the indicated plants were inoculated with Fo5176 spores. Disease symptoms were photographed and analyzed 15 days after inoculation. The analysis of disease severity was conducted based on the count of plantlets exhibiting varying degrees of disease. The disease grading was established on the following scale: grade 1 indicated plants exhibiting only minor growth impairment compared to untreated controls; grade 2 indicated stunted growth and a generally compromised health status; and grade 3 indicated plants that were deteriorating towards mortality or had already succumbed

To determine whether MaPTI6L is involved in plant disease resistance, *Alternaria alternata* was used to infect *N. benthamiana* leaves after transient expressing MaPTI6L. MaPTI6L-infiltrated leaves had smaller lesions than GFP-control leaves (Fig. 4A and B). In contrast, *N. benthamiana* leaves co-infiltrated with MaPTI6L and *Foc-*milR87 showed larger lesions in comparison with those expressing MaPTI6L alone (Fig. 4C and D).

We further investigate the effect of MaPTI6L on plant defense against *F. oxysporum*, *Arabidopsis* transgenic lines overexpressing MaPTI6L under the control of the Cauliflower Mosaic Virus 35S promoter were generated. These transgenic plants were subjected to root-dip inoculation with the *F. oxysporum* strain Fo5176, which is known to be pathogenic to *Arabidopsis*. After 15 days of incubation, MaPTI6L-transgenic *Arabidopsis* plants exhibited improved growth, increased survival rate, and milder disease symptoms, when compared to the WT Col-0 plants upon exposure to Fo5176 (Fig. 4E and F). Collectively, the above results indicate that MaPTI6L plays a role in modulating plant resistance to fungal infection.

### Nuclear localization of MaPTI6L is required for activation of plant immunity

**Figure 5:**
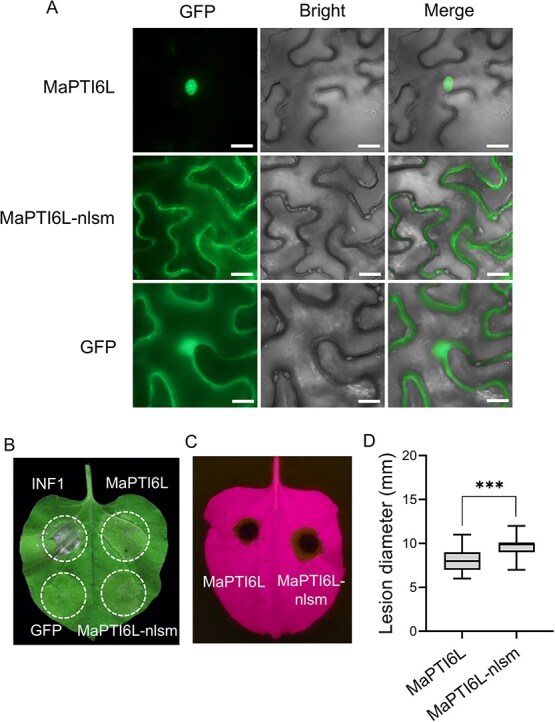
MaPTI6L is located in the nucleus, and the nuclear localization of MaPTI6L is required for its immune function. (A) The subcellular localization of MaPTI6L. GFP was used as control, and fluorescence emitted from the epidermal cells of *N. benthamiana* was visualized under laser scanning confocal microscopy at 2 dpa. (B) Chlorosis triggered by MaPTI6L but not by MaPTI6L-nlsm in *N. benthamiana* leaves. Leaves were infiltrated with *Agrobacterium tumefaciens* carrying pBinGFP::MaPTI6 or pBinGFP::MaPTI6L-nlsm. Images were captured at 5 dpa, utilizing GFP as the negative control and INF1 as the positive control. (C and D) Overexpression of MaPTI6-nlsm does not confer resistance to *A. alternata* in *N. benthamiana* leaves. The error bars represent maximum and minimum values. Center line,median; box limits, 25th and 75th percentiles; *n* = 10. *** indicates *P* < 0.001 (Student’s *t*-test)

To confirm the specific subcellular location of MaPTI6L, the GFP-tagged MaPTI6L was transiently expressed in *N. benthamiana* leaves via agroinfiltration. The results showed that green fluorescence was exclusively confined to the nucleus (Fig. 5A). When the predicted nuclear localization signal site (amino acids 114-123) in MaPTI6L was mutated from RRRP to AAAA, creating the mutated MaPTI6L-nlsm, no nuclear green fluorescence was observed (Fig. 5A), indicating that MaPTI6L indeed localizes specifically in the nucleus.

To test the effect of subcellular localization of MaPTI6L on plant immunity, both MaPTI6L and its mutated form MaPTI6L-nlsm were transiently expressed in *N. benthamiana* leaves before challenging them with *A. alternata*. Unlike MaPTI6L, the expression of MaPTI6L-nlsm did not induce chlorosis or cell death within 48 hpa (Fig. 5B). Notably, when infected by *A. alternata*, leaves expressing MaPTI6L-nlsm developed larger necrotic lesions compared to those expressing MaPTI6L (Fig. 5C and D). These findings suggest that the nuclear localization of MaPTI6L is critical for triggering plant immunity and conferring disease resistance.

### MaPTI6L promotes the expression of *MaEDS1*, a key gene associated with SA signaling pathway

**Figure 6:**
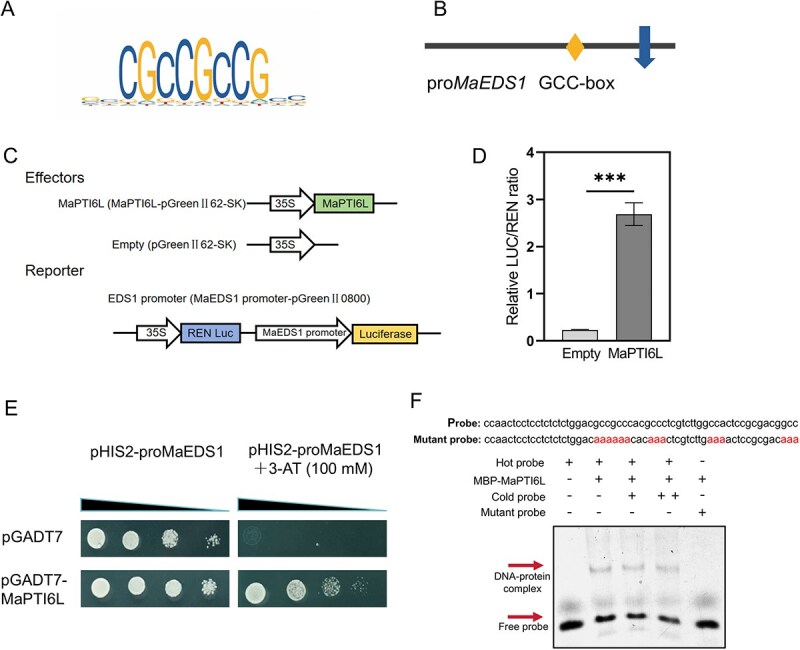
MaPTI6L targets the promoter of *MaEDS1* and positively regulates its expression. (A) Potential binding elements of *MaPTI6L* identified by JASPAR. (B) *MaEDS1* promoter sequence analysis revealed the presence of GCC-box binding element. (C) Schematic diagram of effectors and reporter, and constructs used in the dual-LUC reporter assays in *N. benthamiana* leaves. MaPTI6L and REN Luc were driven by the 35S promoter; LUC was driven by the *MaEDS1* promoter. (D) Leaf discs were collected for LUC activity measurements. Data are shown as means ± SD (*n* = 10). *** indicates *P* < 0.001 (Student’s *t*-test). (E) Y1H assay revealed MaPTI6L binding to *MaEDS1* promoter. The yeast strain co-expressed with pGADT7 and pHIS2-pro*MaEDS1* was used as the control. (F) Purified recombinant protein MBP-MaPTI6L binds to the *MaEDS1* promoter in EMSA assay

Our previous transcriptomic analysis highlighted *MaEDS1* as one of the most significantly up-regulated genes in response to infection with the Δ*Foc-*milR87 mutant (Fig. S5). An online prediction by JASPAR revealed that MaPTI6L could bind GCC-box cis-acting regulatory element, which was also found in the promoter region of *MaEDS1* (Fig. 6A and B). Thus, *MaEDS1* was chosen as a representative candidate gene for further investigation. We further performed qRT-PCR to detected the expression levels of the *N. benthamiana* endogenous *EDS1* after MaPTI6L was transiently expressed. The results showed that the endogenous *NbEDS1* was also induced in *N. benthamiana* (Fig. S6A and B)*.* Using the luciferase (LUC) reporter system, we sought to test if MaPTI6L could regulate the expression of *MaEDS1* by interacting with its promoter. To this end, we cloned a 2-kb fragment of the selected *MaEDS1* promoter up to the translation start codon upstream of the LUC gene to generate the construct pro*MaEDS1*::LUC. Compared to the empty control, the LUC activity was significantly higher in leaves co-infiltrated with both pro*MaEDS1*::LUC and MaPTI6L (Fig. 6C and D). Furthermore, the yeast-one-hybrid (Y1H) assay revealed that yeast strain carrying both pGADT7-MaPTI6L and pHIS2-*proMaEDS1* plasmids successfully grew on medium supplemented with 100 mM 3-amino-1, 2, 4-triazole (3-AT). Conversely, the yeast strain harboring only the pHIS2-*proMaEDS1* construct could not survive under the same conditions (Fig. 6E). *In vitro* electrophoretic mobility shift assays (EMSA) also demonstrated that MaPTI6L binds to the 40 bp fragment harboring the core GCC-box in *MaEDS1* promoter, but not the GCC-box mutant probe (Fig. 6F). These above results strongly imply that MaPTI6L promotes the transcription of *MaEDS1* by directly binding to its promoter region.

It is documented that EDS1 plays an essential role in regulating SA biosynthesis or response-related genes [30]. We therefore assessed the SA content in bananas inoculated with the WT and the Δ*Foc*-milR87 mutant. As anticipated, the SA content was significantly higher in banana inoculated with the Δ*Foc*-milR87 mutant at 24 and 48 hpi (Fig. S7), indicating that *Foc*-milR87 may inhibit the expression of *MaEDS1* by silencing *MaPTI6L*, and ultimately inhibit the SA immune pathway in banana.

### The *Foc*-milR87 target site in the 3'UTR of *MaPTI6L* is critical for plant susceptibility

**Figure 7:**
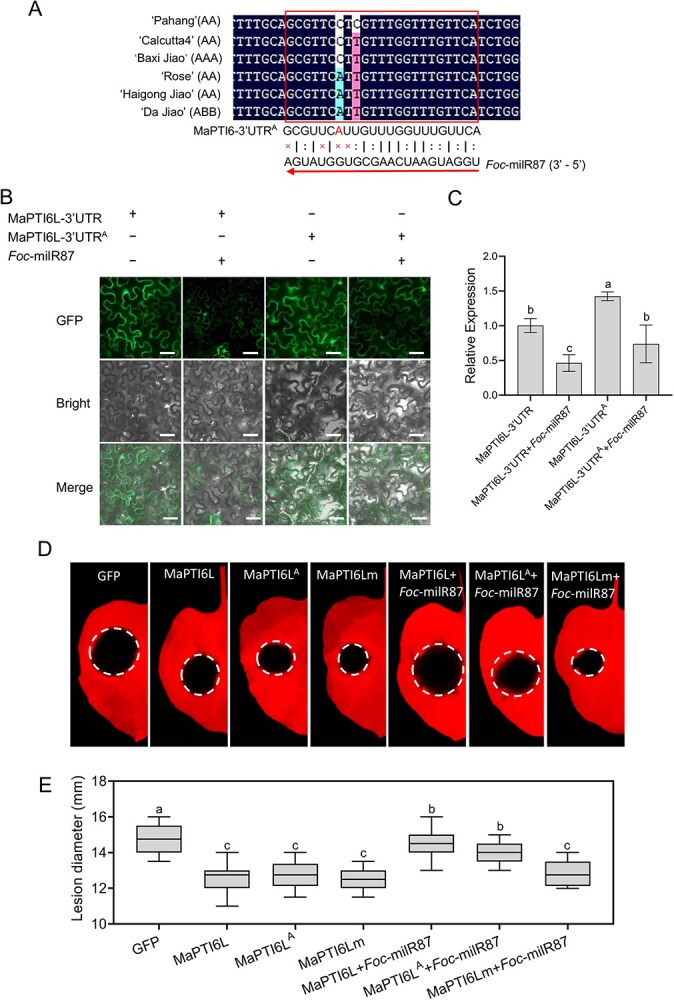
*Foc*-milR87 target site in 3'UTR is critical for disease resistance of MaPTI6L. (A) Gene sequence alignment of *MaPTI6L* in different banana varieties. (B) Co-infiltration of MaPTI6L-3'UTR or single nucleotide mutated MaPTI6L-3'UTR^A^ with *Foc*-milR87 precursor in *N. benthamiana*. Confocal images were taken at 48 h after agro-infiltration. Bar, 50 μm. (C) Transcript levels of *GFP* in different treatments were detected by qRT-PCR and were normalized to the treatment with GFP-marked MaPTI6L-3'UTR alone. (D and E) Mutation in the 3'UTR of *MaPTI6L* conferred *N. benthamiana* resistance against *A. alternata*. Photographs were taken at 5 dpa. A Duncan’s multiple range test was used for significant analysis. The error bars represent maximum and minimum values. Center line,median; box limits, 25th and 75th percentiles.; *n* ≥ 8

Sequence alignment of the *MaPTI6L* gene in 19 banana varieties revealed the presence of two SNPs in the 3'UTR of its mRNA (Fig. S8). One SNP, a C-to-A mutation, was identified in disease-resistant or tolerant varieties such as 'Rose', 'Haigong Jiao', 'Da Jiao', as compared to susceptible Cavendish cultivar ‘Baxi Jiao’, and two cultivars with published genomes, 'PaHang' and 'Calcutta4'. The other SNP, a prevalent C-to-T mutation, was found in the majority of banana varieties, including cultivated 'Baxi Jiao' and wild type varieties like 'Rose', 'Haigong Jiao', 'Da Jiao', and 'Calcutta4'. These SNPs are situated precisely at the binding site of *Foc*-milR87 (Fig. 7A; Fig. S8), potentially altering *Foc*-milR87 recognition and thereby affecting MaPTI6L-mediated plant resistance to *Foc* TR4.

Given the presence of C-to-A mutation in resistant varieties, a construct harboring this SNP, designated MaPTI6L-UTR^A^, was created using the established method for identifying *Foc*-milR87 target sites. The results demonstrated significantly higher GFP expression from the MaPTI6L-UTR^A^ construct compared to MaPTI6L-UTR. Although *Foc*-milR87 reduced the GFP signal, the suppression rate of MaPTI6L-UTR^A^ was not more pronounced than that of MaPTI6L-UTR (Fig. 7B and C). To assess the impact of 3'UTR mutations on MaPTI6L-conferred disease resistance, *N. benthamiana* leaves were infiltrated with MaPTI6L-UTR, MaPTI6L-UTR^A^, or MaPTI6L-UTRm before inoculation with *A. alternata*. Consistent with expectations, leaves expressing MaPTI6L-UTR, MaPTI6L-UTR^A^, or MaPTI6L-UTRm developed smaller lesions compared to the GFP control (Fig. 7D, E). Subsequently, when co-expressed with *Foc*-milR87 and inoculated with *A. alternata*, *N. benthamiana* leaves expressing MaPTI6L-UTR^A^ displayed lesion sizes that, albeit not statistically significant, were marginally reduced compared to those expressing MaPTI6L-UTR; notably, lesions in plants expressing MaPTI6Lm were the smallest (Fig. 7D and E). And the collective results indicated that the *Foc*-milR87 target site in the 3'UTR plays the crucial role in MaPTI6L-mediated plant susceptibility.

## Discussion

Based on our previous research, we identified a FoQDE2-dependent milRNA, *Foc*-milR87, which acts as a virulence effector by targeting mRNA of glycosyl hydrolase (GH79C) in *Foc* [25]. In this study, we discovered that *Foc-*milR87 is highly conserved and specific in the *Fusarium* species, including *F. oxysporum* species complex (FOSC), *F. fujikuroi* and *F. proliferatum*, with identical gene loci in the intergenic regions. The mature milRNA sequences homologous to *Foc*-milR87 are exactly the same, including a recently identified *Fol-*milR1, a virulence factor in *F. oxysporum* f. sp. *lycopersici* that impairs host immunity by targeting a CBL-interacting protein kinase coding gene *SlFRG4* [16]. The above results indicated that *Foc*-milR87 is restricted to *Fusarium* species and is a typical milRNA that functions as a virulence effector during fungal infection processes.

Recent researches have revealed that milRNAs can silence the host plant target genes via cross-kingdom RNAi [31, 32], thereby playing a critical role in the interactions between plants and pathogens. In this study, we prove that the cross-kingdom RNAi also exists in the interaction process between *Foc* and bananas. In target gene identification assays employing the *N. benthamiana* transient expression system, *Foc*-milR87 was highly expressed and capable of decreasing the mRNA and protein abundance of MaPTI6L (Fig. 2F and G). However, in *Foc*–banana interaction system, qRT-PCR results showed that *MaPTI6L* is slightly induced by *Foc* at the initial infection stage (6–72 hpi), and substantially induced by Δ*Foc-milR87* infection at the same period (Fig. S2). It is worth noting that high auxin condition can trigger higher accumulations of mature miR408-5p, thereby leading to switch the miRNA action from translation repression to mRNA cleavage in rice [33]. Based on this finding, we speculate that higher accumulations of *Foc*-milR87 leads to the cleavage of *MaPTI6L* mRNA, resulting in significant decrease in mRNA and protein levels. While, during the early infection stages, the levels of *Foc*-milR87 are low, it may inhibit target gene translation rather than induce its mRNA cleavage. We attempted to identify potential cleavage sites of *MaPTIL6* mRNA using the 5' RNA ligase-mediated rapid amplification of cDNA ends (5′ RLM-RACE) assay [34], but were unsuccessful. This failure may be attributed to the challenges in detecting cleavage sites at the 3' UTR region or the possibility that *Foc*-milR87 inhibits translation rather than inducing mRNA cleavage. Therefore, the precise mechanism of action mode of *Foc*-milR87 requires further investigation.

Interacting proteins of resistance protein Pto (PTIs), including SlPTI1, SlPTI4, SlPTI5 and SlPTI6, were first identified in tomato [26, 35]. SlPTI1, a Pto-phosphorylated serine/threonine kinase, is involved in the ETI defense responses to *Pseudomonas syringae* pv. *tomato* (*Pst*) [36], while SlPTI4, SlPTI5, and SlPTI6 belong to the AP2 transcription factor family and are able to bind with Pto, thereby playing a pivotal role in plant immunity [17–20]. SlPTI4 and SlPTI5, which are induced by the bacterial speck pathogen *Pst*, play crucial roles in plant disease resistance [26, 37]. Furthermore, overexpression of SlPTI4, SlPTI5, and SlPTI6 in *Arabidopsis* and *Solanum lycopersicum* triggers defense responses [26, 37–39]. However, SlPTI6 is not significantly induced by *Pst* infection, SA, or ethylene in tomato [37, 40]. Transgenic *Arabidopsis* plants expressing SlPTI6 failed to improved resistance against *Erysiphe* sp. or tolerance to *Pst*, indicating that disease resistance mechanism regulated by SlPTI6 are different from those of SlPTI4 and SlPTI5 [37, 41]. In this study, MaPTI6L was phylogenetically distant from SlPTI6 identified in tomato, suggesting MaPTI6L may has a different disease resistance mechanism. The transcription of *MaPTI6L* is substantially induced by Δ*Foc-milR87* infection at the initial infection stage (6–72 hpi), indicating that MaPTI6L, modulated by *Foc*-milR87, is positively regulating resistance to *Foc*.

**Figure 8:**
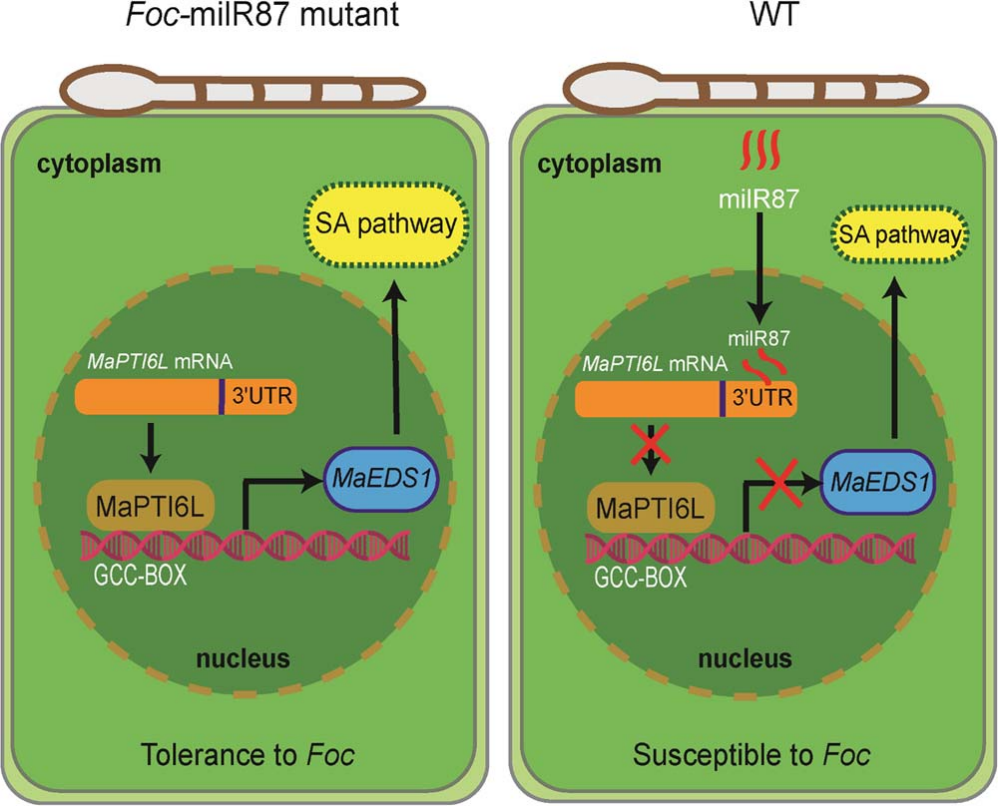
A proposed model illustrating the virulence mechanism of *Foc*-milR87. MaPTI6L, an AP2 family transcription factor of banana, can activate the expression of a subset of defense-related genes in response to pathogen infection. MaPTI6L can bind to the GCC-box in the promoter region of *MaEDS1* and activate its expression. However, during *Fusarium* infection, *Foc*-milR87 enters banana cells and specifically binds to the 3'UTR region of *MaPTI6L* mRNA to promote the degradation of *MaPTI6L* mRNA, ultimately suppressing the expression of *MaEDS1*

An array of pathogenesis-related (PR) genes can be induced by the transcription factors SlPTI4, SlPTI5, and SlPTI6 via specific interaction with the GCC-box cis-acting elements and display distinct regulatory patterns [41]. Specifically, SlPTI4 in *Arabidopsis* has been shown to activate both SA-responsive genes, such as *PR1* and *PR2*, and those ET/JA-responsive genes, including *PR3*, *PR4*, *PDF1.2*, and *Thi2.1*, all of which characteristically contain GCC-box motif [37]. Similarly, SlPTI6 also enhances the expression of *PR1*, *PR2*, and *Thi2.1* [37]. Grapevine *PTI6* is induced after white rot infection and highly expressed in resistance cultivar ‘Zhuosexiang’, which plays a crucial role in promoting *PR1* expression in white rot resistance [42]. In our study, we observed that overexpression of MaPTI6L stimulates various SA/ET/JA signaling pathway marker genes in *N. benthamiana* as well as enhances resistance against *F. oxysporum* in *Arabidopsis*. We also detected the expression of two banana *PR1* genes and found both of them were up-regulated in banana infection by the Δ*Foc-milR87* mutant (Fig. S9). However, no GCC-box motif was found in their promoter region, which indicating that the MaPTI6L can regulate the expression of resistance genes either directly or indirectly. Notably, we recognized *MaEDS1*, a critical SA signaling pathway marker gene harboring a GCC-box in its promoter, as a novel downstream target of MaPTI6L. Intriguingly, studies have showed that EDS1 facilitates defense activation by physically interacting with NPR1 to recruit it onto the *PR1* promoter [43]. Consequently, these findings imply that MaPTI6L plays a direct role in activating *MaEDS1* and subsequently *PR1* expression to mediate defense responses (Fig. 8).

Resistance against biotrophic and hemibiotrophic pathogens is initiated by SA, whereas defense responses confer resistance to necrotrophic pathogens are predominantly regulated via JA and ethylene (ET)-mediated pathways [44]. Our findings on the function of MaPTI6L indicate it significantly enhanced plant resistance to *A. alternata* and *F. oxysporum*, which are considered as necrotrophic and hemibiotrophic fungi, respectively [45, 46]. Hemibiotrophic pathogens like *F. oxysporum* exhibit a dual infection strategy, initially requiring host cell survival during the biotrophic phase before transitioning to necrotrophic infection characterized by cell death. Therefore, distinct plant hormone pathways may come into play during these different infection stages. Previous research has shown that activation of plant immune genes can engage multiple plant hormone pathways, providing resistance against hemibiotrophic pathogens [47, 48]. SA likely contributes to disease resistance during the initial biotrophic phase, while JA and ET may function during the necrotrophic phase. Given the large number of disease-resistant marker genes that were substantially up-regulated by MaPTI6L overexpression, we speculate that MaPTI6L may regulate banana disease resistance against pathogens belonging to different lifestyles.

Plant–pathogen coevolution drives the emergence of diverse mechanisms of plant defense and pathogen counter-defense. Here, we demonstrated that the sRNA *Foc-*milR87 specifically interacted with the 3'UTR of host *MaPTI6L*, which encodes a transcription factor that positively modulates plant immune responses, to reduce the mRNA accumulation of *MaPTI6L* and facilitate pathogen infection (Fig. 8). On the other hand, the 3'UTR of *MaPTI6L* in several wilt-resistant banana varieties has a SNP, which associated with avoidance of binding by *Foc*-milR87. In nature, abundant evidence showed that pathogen effectors are able to evade the recognition by the plant immune system through nucleotide mutations in the effectors to diversify their expression and subcellular localization [49]. Nevertheless, reports of plant host gene mutations to avoid pathogen effector binding are scarce. The SNPs in the 3'UTR of MaPTI6L allow the host plant to evade recognition by the sRNA effector (*Foc*-milR87) from the pathogen, thereby developing resistance against pathogen infection without altering the protein structure and function. Our findings on SNPs provide more options for applying these resistance genes in crop breeding for disease resistance. By modifying only the UTR region, rather than the entire coding region, transgenic plants are avoided, which improves banana disease resistance breeding in a more acceptable and sustainable manner.

### Conclusion

This study reveals an intricate mechanism of fungal pathogenesis in which *Foc* employs the sRNA effector *Foc*-milR87 to impair host plant immune responses by targeting the 3'UTR of *MaPTI6L*, a pathogenesis-related gene in bananas that encodes a AP2 transcription factor. Functional analysis of MaPTI6L indicates its direct role in activating plant disease resistance through the SA signaling pathway, as evidenced by its promotion of *MaEDS1* expression. Notably, SNPs detected in the 3'UTR of *MaPTI6L*, especially in Fusarium wilt-resistant banana varieties, significantly reduce the effectiveness of *Foc*-milR87 in suppressing target gene expression. These findings highlight the potential of targeting the 3'UTR of *MaPTI6L* for genome editing to develop disease-resistant banana cultivars. In summary, this research advances our understanding of fungal milRNAs in host-pathogen interactions, and opens new avenues for enhancing plant disease resistance.

## Materials and methods

### Fungal strains and conidiation conditions

The *F. oxysporum* f. sp. *cubense* tropical race 4 strain XJZ2, isolated by Li et al. [50], was used in the experiments. The *F. oxysporum* strain 5176 [51] was used for Arabidopsis plants inoculation. The plant pathogen *A. alternata* isolated from tomato was used to detect disease resistance. The induction of conidiation followed the procedure described in our previous study [52].

### Plant materials and cultivation conditions

*N. benthamiana* plants and tissue-cultured plantlets of the Cavendish banana cv 'Baxi Jiao' were cultivated in growth chambers with an ambient temperature of 26°C under a 16-h day/8-h night photoperiod. Arabidopsis plants were grown at 23°C with a 10-h light/14-h dark photoperiod. Additional banana varieties exhibiting resistance or tolerance to *Fusarium* wilt, notably 'Rose', 'Haigong Jiao', and 'Da Jiao' [53, 54], were kindly supplied by Professor Bingzhi Huang from the Institution of Fruit Tree Research, Guangdong Academy of Agricultural Sciences.

### Nucleic acid manipulation, milRNA detection, and qRT-PCR analysis

Total RNA from banana infected with *Foc* was extracted using CTAB (Dingguo, China). cDNA was synthesized and gene expression was detected using qRT-PCR as previously reported [52]. For milRNA expression, PolyA polymerase (NEB, USA) was used to introduce PolyA tails to total RNAs. MilRNA was then reverse-transcribed using an oligo-dT adaptor. The transcript abundance of milRNA was detected using qRT-PCR, as described previously [25]. Total RNA from *N. benthamiana* leaves was isolated using RNA Mini-preps Kit (Bio Basic, Canada) according to manufacturer’s protocol. The expression levels of several defense-associated genes, including *NbPR1*, *NbPR4*, *NbLOX*, *NbERF1*, *NbOsmotin*, *NbACCO1*, *NbHir1*, and *NbHin1* [55–57], were assessed through qRT-PCR using *NbEF1*α gene as reference. A minimum of three biological replicates were required for each sample.

### Phylogenetic analysis and multiple sequence alignment

Using the precursor sequence of *Foc*-milR87 as a query, we conducted the default BLASTN searches against the genomic sequences in the ENSEMBL fungal database (https://fungi.ensembl.org/Tools/Blast?tl=Kl7PcOATzPbMf4XF-21631558). Homologs exceeding 50 bp in length were retained for phylogenetic analysis. Homologous proteins were searched by running BLASTP against the NCBI database (https://blast.ncbi.nlm.nih.gov/Blast.cgi). The conserved domain of PTI6 proteins was predicted online (https://www.ncbi.nlm.nih.gov/Structure/cdd/wrpsb.cgi). Multiple sequence alignment was conducted by DNAMAN (version 7) with default parameter settings. A phylogenetic tree was generated using the maximum likelihood approach as outlined earlier [58], with MEGA-X software package and a bootstrap replication count of 1000.

### Protoplast preparation and total RNA extraction of banana roots in response to *Foc* infection

Freshly grown banana roots were inoculated with *Foc* conidial suspension at a concentration of 1 × 10^8^ spore ml^−1^, and mock inoculated with water. Post a 36-h incubation period, the root tips were harvested and minced into fine pieces (2 mm in size). These minced root tips were immersed in an enzyme solution formulated as described previously [59]. The mixture was incubated for 3 h at 26°C with gentle shaking and the undigested root debris in cellular suspension were eliminated through a 40-μm filter. The filtrate was gently washed with 15 ml washing buffer, followed by centrifugation at 200 g for 5 min at 4°C. The supernatant about half volume was carefully removed, and the rinsing step was repeated. Finally, total RNA was extracted from the protoplasts using Trizol reagent.

### *Foc-*milR87 target gene prediction and identification

The target genes of *Foc*-milR87 in the banana genome (https://banana-genome-hub.southgreen.fr/) were predicted using the online program psRNATarget (https://www.zhaolab.org/psRNATarget/). The expect alignment score was set at ≤4.5. Then gene expression values of these predicted genes assessed by qRT-PCR. Compared with infection by the WT strain XJZ2, the genes with substantially up-regulated expression in Cavendish cultivar ‘Baxi Jiao’ [25] in response to infection by the mutant Δ*Foc-milR87* were selected as candidates for target site identification. If the target site of *Foc*-milR87 was located at the 3'UTR of the candidate target gene, only the 3'UTR of *MaPTI6L* was inserted into the 3' terminal of GFP. Additionally, the mutated 3'UTRs (MaPTI6L-3'UTRm representing for multi-locus mutations and MaPTI6L-3'UTR^A^ denoting a single nucleotide mutation observed in disease-resistant varieties) were also introduced into the plasmid pBinGFP for target site identification using the method described previously [15].

### Plasmid construction

For pBinGFP::*MaPTI6L*-3'UTR/-3'UTR/-3'UTRsm construction, the primers (S1 Table) with *Sma*I and *Bam*HI enzyme restriction site were designed according to the 3'UTR sequence of *MaPTI6L*. And then the 3'UTR sequence was amplified and introduced into the vector pBinGFP, while the plasmids for milRNA and target gene expression were constructed adopting the method described previously [25].

### Transient expression in *N. benthamiana* mediated by *agrobacterium*

For transient expression, *A. tumefaciens* GV3101 carrying the recombinant vector were infiltration into *N. benthamiana* leaves adopting the procedure described previously [59]. The treated leaves were photographed and then collected for detecting mRNA and protein levels of GFP, MaPTI6L, as well as transcript levels of disease-resistant marker genes 48 h after infiltration [55–57], which have been described previously in *N. benthamiana*.

### Protein extraction and western blot assay

Protein extraction and western blot analysis were conducted as outlined [59]. Briefly, *N. benthamiana* leaves were ground in liquid nitrogen and poured into a 2-ml RNase-free centrifuge tube containing 1 ml of protein extraction buffer. The mixture was vortexed, incubated on ice for 10 min, and centrifuged at 13 523 g at 4°C for 10 min to obtain the protein supernatant. Total proteins were separated by SDS-PAGE and transferred to PVDF membranes (Bio-Rad, USA). Western blotting was performed using a 1:3000 dilution of mouse anti-GFP monoclonal antibody (Abmart, China) and a 1:10 000 dilution of goat anti-mouse antibody (Dingguo, China). Protein bands were visualized using an Efficient Chemiluminescence kit (Genview, China). And imaging was recorded using the ChemDoc XRS imaging system (Bio-RAD, USA).

### Reactive oxygen species and callose staining

For the reactive oxygen species (ROS) accumulation assay, *N. benthamiana* leaves were collected 36 h post-*Agrobacterium* infiltration and submerged in 3,3′-diamino benzidine staining solution with the abaxial side facing down. The leaves were incubated in the dark at 26°C for 12 h followed by boiling in anhydrous ethanol for 15 min to decolorize. The leaves were then dried and photographed. For the callose deposition analysis, leaf samples were dipped abaxially downward in aniline blue staining solution and kept in the dark for 2 h [60]. Imaging was recorded using an Olympus BX53 microscopy system. The ROS burst and callose deposition were quantified by ImageJ software, with a minimum of three leaves examined per experiment.

### Disease resistance detection

The pathogen, *A. alternata*, isolated from tomato was cultured on PDA at 25°C. After one week, 3-mm-diameter mycelium block were put on the abaxial side of *N. benthamiana* leaves. Then, the inoculated detached tobacco leaves were placed at 25°C on moisturized filter paper inside covered boxes, and the lesion area were measured 5 days later. In each treatment, at least ten leaves were inoculated.

### Assay for confocal microscopy images

For fluorescence observations, 5-mm-diameter leaves pieces of *N. benthamiana* were excised and subjected to confocal imaging utilizing Nikon A1 laser scanning microscope (Nikon, Japen). GFP fluorescence was detected under an excitation wavelength of 488 nm.

### Analysis of *cis*-acting regulatory element

The promoter regions (2000 bp upstream of the translation start codon) of the target genes were extracted from the banana genome [61] and uploaded to the PlantCARE website (http://www.bioinformatics.psb.ugent.be/webtools/plantcare/html/) for the computational identification of various known cis-acting elements using default settings [62].

### Y1H assay

The promoter sequence of *MaEDS1* was amplified via PCR. The amplicon was cloned into the pHIS2 vector to yield the bait construct, *proMaEDS1*::HIS3. Separately, the coding sequence of *MaPTI6L* was inserted into the pGADT7 vector to generate the prey construct pGADT7::MaPTI6L. To suppress background growth, 3-amino-1, 2, 4-triazole (3-AT) was used at appropriate concentrations. Subsequently, the above recombinant vectors were cotransformed into the yeast strain Y187 as described previously [63]. The transformants were evaluated by spotting them on synthetic defined (SD) media without Trp, Leu, and His, and supplemented with 100 mM 3-AT. Yeast growth was observed after incubation at 28°C for 48 h to assess protein-DNA interaction.

### Dual-LUC assays

To construct the 35S::MaPTI6L effector recombinant vector, the coding sequence of *MaPTI6L* was inserted into vector pGreenII 62-SK. The promoter region of *MaEDS1* was inserted into vector pGreenII 0800-LUC to create the reporter construct, *proMaEDS1*::LUC. For the infiltration assay, Agrobacterium strain AH105 (Weidi Biotechnology, China) harboring the indicated vectors were resuspended in infiltration buffer and infiltrated into the *N. benthamiana* leaves. At 48 h post-infiltration, leaf discs from *N. benthamiana* were harvested for the LUC and Renilla LUC (REN) activities detection using a dual-LUC reporter assay kit (Beyotime, China). Each experiment was performed with three separate biological replicates.

### EMSA

The CDS sequence of *MaPTI6L* was amplified and cloned into the pTAG2K expression vector, and then the fusion vector was expressed in the DE3 strain of *Escherichia coli*. Recombinant MBP-MaPTI6L proteins were purified following the manufacturer’s instructions provided by Smart-Lifesciences (China). Fam-labeled probes, along with their mutant counterparts, were synthesized by Youkang (China). Specific binding interactions were verified using 100-fold unlabeled and sequence-identical oligonucleotides to compete the labeled probes. EMSA analysis was conducted according to the protocols outlined in the EMSA Kit (Beyotime, China), with bands visualized as per the manufacturer’s recommendations.

## Supplementary Material

Web_Material_uhae361

## Data Availability

All data in this study were provided in the article and its supplementary materials.
