# Supplementary material for: A virulent milRNA of Fusarium oxysporum f. sp. cubense impairs plant resistance by targeting banana AP2 transcription factor coding gene MaPTI6L
Source: Hortic Res. 2024 Dec 28;12(4):uhae361. doi: 10.1093/hr/uhae361 (PMC11894533; doi:10.1093/hr/uhae361)
Supplement: Web_Material_uhae361 [file web_material_uhae361.zip › Supplemental Information.doc]

**Figure S1.** Expression patterns of *Foc*-milR87. Total RNAs were extracted from the host banana roots inoculated with the *Foc* wild type strain XJZ2 at the different time point (0-96 h prior). Relative expression levels of *Foc*-milR87 were calculated by the 2^-ΔΔCt^ method using snRNA (U4) of *Foc* as internal control. Error bars indicate S.D; n =3.

**Figure S2.** Transcriptional pattern analysis of *MaPTI6L* in response to infection by the wild type XJZ2 and the *ΔFoc-milR87* mutant of *Foc*. Relative expression levels of *MaPTI6L* were calculated by 2^-ΔΔCt^ method using the *Actin* gene from banana as internal control. A Student’s *t*-test was used to identify significant differences between samples inoculated with XJZ2 and the *ΔFoc-milR87* mutant. *** indicates p<0.001, ** indicates p<0.01, * indicates p<0.05.

**Figure S3.** Expression of *MaPTI6L* silenced by *Foc*-miR87. Relative expression levels of *MaPTI6L* (CDS + 3'UTR) and its 3'UTR mutant MaPTI6Lm in *N. benthamiana* was detected after agro-infiltration at 48 hpa by qRT-PCR. The constitutively expressed gene *NbEF1α* was used as internal reference. Mean SD values were calculated from three technical replicates. A Student’s *t*-test was used to identify significant differences between samples with or without *Foc*-milR87. *** indicates p<0.001. Similar results were obtained from three biological replicates.

**Figure S4.** The conserved domain and phylogenetic analysis of MaPTI6L. (A) Conserved domain of MaPTI6L was predicted, and the orange bar represents AP2 with green triangles representing DNA binding site (DBS), and the orange triangle representing nuclear localization signal site (NLS). (B) The maximum likelihood tree was constructed using MEGA X. Bootstrap percentages out of 1000 replications are shown at branch points. The target MaPTI6L is shown in blue font.

**Figure S5.** The transcriptome data of *MaPTI6L* and *MaEDS1* in bananas in response to infection by the wild type XJZ2 and the Δ*Foc-milR87* mutant of *Foc* at the different time points (0h, 24h, 48h). Expression levels are normalized to the mean Fragments Per Kilobase of transcript per Million mapped reads (FPKM) value across all time points.

**Figure S6.** Protein sequence alignment of MaEDS1 and its orthologs and the relative expression levels of *MaEDS1* in MaPTI6L-expressing leaves. (A) Protein sequence alignment of MaEDS1 and its orthologs from *N. benthamiana* and *Arabidopsis.* Ma: banana; Nb: *N. benthamiana*; At: *Arabidopsis.* Columns with identical and similar amino acid sequences were colored black and gray, respectively. (B) Transient expression of MaPTI6L in *N. benthamiana* leaves activates the expression of *NbEDS1*. The expression of *NbEDS1* was detected at 48 hours post-agroinfiltration by qRT-PCR*.* The constitutively expressed gene *NbEF1α* was used as internal reference. Mean SD values were calculated from three technical replicates. Similar results were obtained from three biological replicates.

**Figure S7.** The SA production of banana in response to infection by the wild type strain XJZ2 and the Δ*Foc*-milR87 mutant of *Foc*. Data represent means ± SE from 3 independent biological repeats. A Student’s *t*-test was used to identify significant differences between samples inoculated with XJZ2 and the Δ*Foc*-milR87 mutant. *** indicates p<0.001, ** indicates p<0.01, ns indicates no significant difference.

**Figure S8.** Gene sequence alignment of *MaPTI6L* in 19 banana varieties. Red frame indicated the milR87 target site.

**Figure S9.** Transcriptional analysis of *MaPR1a* and *MaPR1b* in response to infection by the wild type XJZ2 and the *ΔFoc-milR87* mutant of *Foc*. (A, B) Relative expression levels of *MaPR1a* and *MaPR1b* were calculated by 2^-ΔΔCt^ method using the *Actin* gene from banana as internal control. A Student’s t-test was used to identify significant differences between samples inoculated with XJZ2 and the *ΔFoc-milR87* mutant.** indicates p<0.01, * indicates p<0.05, ns indicates no significant difference.

**Table S1 primers used in the study**

| **Primer name** | **Sequences(5'-3')** | **Purpose** |
| --- | --- | --- |
| qGFP-F | ATCATGGCCGACAAGCAGAA | qRT-PCR |
| qGFP-R | AACTCCAGCAGGACCATGTG |  |
| qNbEF1α-F | TTGCTTGCTTTCACCCTTGG |  |
| qNbEF1α-R | TCGAAACCAGAGATGGGGAC |  |
| qMusa-β-actin-F | GGTATCGTGTTGGATTCTGGA |  |
| qMusa-β-actin-R | GTAGTCTCATGGATACCTGC |  |
| qPTI6-F | GTAACTCAAAAGCCCTTCGG |  |
| qPTI6-R | CCTGACTCCCTTAAACTTGG |  |
| qNbPR1-F | CCGCCTTCCCTCAACTCAAC |  |
| qNbPR1-R | GCACAACCAAGACGTACTGAG |  |
| qNbPR2-F | AGGTGTTTGCTATGGAATGC |  |
| qNbPR2-R | TCTGTACCCACCATCTTGC |  |
| qNbPR4-F | GGCCAAGATTCCTGTGGTAGAT |  |
| qNbPR4-R | CACTGTTGTTTGAGTTCCTGTTCCT |  |
| qNbLOX-F | AAAACCTATGCCTCAAGAAC |  |
| qNbLOX-R | ACTGCTGCATAGGCTTTGG |  |
| qNbERF1-F | GCTCTTAACGTCGGATGGTC |  |
| qNbERF1-R | AGCCAAACCCTAGCTCCATT |  |
| qNbPAL4-F | TTACGCCCTTCGAACATCCC |  |
| qNbPAL4-R | GGCCTGGCAAAGTGCTACTA |  |
| qNbOsmotin-F | TTCAATGCTGCAGGTAGGGG |  |
| qNbOsmotin-R | TTCCAGGCATTTCCAAGGGG |  |
| qNbACCO1-F | GTGGCCAGTAACGGTCTTGA |  |
| qNbACCO1-R | TCCGAGTCCCGTCTTTTTGG |  |
| qNbHir1-F | TCAGGATGTGCTTGAGCCAG |  |
| qNbHir1-R | TACTTAGACTCGGCCTCCCC |  |
| qNbHin1-F | ATGCCACGTCATAATCCCGT |  |
| qNbHin1-R | AATCTTCAACGTCCTATGACCTGA |  |
| UP2 | CTACGTAACGGCATGACAGTG |  |
| U4-3 | GGGAATTTTTGGAACTCTTT |  |
| qFoc-milR87 | GATGAATCAAGCGTGGTATG |  |
| MaPTI6-3'UTR-F1 | GGATCCTCTAGATGAACTAGATTGACTGGAAATTTTTGCAG | Construction pBinGFP::MaPTI6-3'UTR |
| MaPTI63'UTR-R1 | AGATCAAGCCGAATGACTATCGCTGCAAAATGGACCCTCCTCT |  |
| MaPTI6-3'UTRm-R | TCTAGTTCATCTAGAGGATCCAGAGCTAAGGACAAGCGTCTCATTCACTAAGGGGCGGCTCTTAGAGCGCCAGAAGATCAAGCCGAATGACTATCGCTGCAAAATGGACCCTCCTCT | Construction pBinGFP::MaPTI6-3'UTRm |
| MaPTI6-3'UTRm-F | TGGACGAGCTGTACAAGTAATGTTAGGCTGGAGTCAGTAATCAGGTACTGTGCGCAACA |  |
| MaPTI6-F | ACGATAGCCGGTACCCCCGGGATGAAGCAGGATAAGATGGGG | Construction pBinGFP::MaPTI6 |
| MaPTI6-R | GCCCTTGCTCACCATCCCGGGGAAGTCAAAGTCCATCCACC |  |
| MaPTI6nls-F | GTGGTATAATGTCTAAGTTGTGAGGCTTACAATTTCCATTCGCCA | Construction pBinGFP::MaPTI6nls |
| MaPTI6nls-R | CCATTTCCCCCACGCCGCCGCCGCGACTCCCTTAAACTTG |  |
| Foc-milR87-F | ACGATAGCCGGTACCCCCGGGCTACCTTTATATCGTATCACGCG | Construction pBin::*Foc*-milR87 |
| Foc-milR87-R | AGATCAAGCCGAATGACTATCGCCCTCAGCCTGCAAGACGATTC |  |
| pBinGFPyz-F | GCATTCTACTTCTATTGCAGC | Validation of vector expression |
| pBinGFPyz-R | CTGAACTTGTGGCCGTTTACG |  |
| MaPTI6-3'UTRsm-R | TCTAGTTCATCTAGAGGATCCAGAGCTAAGGACAAGCGTCTCATTCACTAAGGGGCGGCTCTTAGAGCGCCAGATGAACAAACCAAACAATGAACGC | Construction pBinGFP::MaPTI6-3'UTRsm |
| MaPTI6-F | GTAACTCAAAAGCCCTTCGG | qRT-PCR |
| MaPTI6-R | CCTGACTCCCTTAAACTTGG |  |
| LOC103968726-F | GGAAGGAAACCCAAAAACTCC |  |
| LOC103968726-R | CCTGACCCCCTTAAACTTGAC |  |
| LOC103987395-F | GAGAGCAGCAGGAGGAACAA |  |
| LOC103987395-R | CTGACGCCCTTAAACCTGAC |  |
| LOC103990110-F | GACCCGATCGGTAAAACCCT |  |
| LOC103990110-R | GGACGCCCTTGTATCTGCCA |  |
